# Crabgrass as an equine pasture forage: impact of establishment method on yield, nutrient composition, and horse preference

**DOI:** 10.1093/tas/txac050

**Published:** 2022-04-22

**Authors:** Jennifer R Weinert-Nelson, William A Meyer, Carey A Williams

**Affiliations:** Department of Animal Sciences Rutgers, Rutgers, The State University of New Jersey, New Brunswick, NJ 08901, USA; Department of Plant Biology, Rutgers, The State University of New Jersey, New Brunswick, NJ 08901, USA; Department of Animal Sciences Rutgers, Rutgers, The State University of New Jersey, New Brunswick, NJ 08901, USA

**Keywords:** interseeding, summer slump, warm-season grass

## Abstract

Warm-season grasses (WSG) incorporated into traditional cool-season rotational grazing systems to increase summer yields are typically established in monoculture in separate pasture areas. Few studies have evaluated alternative interseeded establishment of WSG, despite potential benefits for improving biodiversity and land-use efficiency. The objective of this study was to determine the impact of establishment method (monoculture vs. interseeded) on crabgrass pasture forage yield, nutritive value, and preference under equine grazing. Three adult standardbred mares grazed two main plots on two consecutive days (8 hr/d) for three grazing events in 2019: Jul 28/29 (GRAZE 1), Aug 20/30 (GRAZE 2), Oct 1/2 (GRAZE 3). Each main plot contained four replicates of three treatments: mixed cool-season grass (CSG); Quick-N-Big crabgrass (CRB) [*Digitaria sanguinalis* (L.) Scop.] interseeded into existing cool-season grass (INT), and CRB established as a monoculture (MON). The cool-season grass mix included *Inavale* orchardgrass [*Dactylis glomerata* (L.)], *Tower* tall fescue [*Lolium arundinaceum* (Schreb.) Darbysh.], and *Argyle* Kentucky bluegrass [*Poa pratensis* (L.)]. Herbage mass (HM) and sward height (SH) were measured prior to each grazing event and samples were collected (0800–1000 h) for chemical composition analysis. Observed grazing time (GT) in each sub-plot as determined by 5-min scan sampling was utilized as marker of horse preference. Forage HM was greater in MON (8043 ± 1220 kg/ha) than CSG (5001 ± 1308 kg/ha; *P* = 0.003), with a trend for greater total HM in MON vs. INT (6582 ± 1220 kg/ha: *P* = 0.06), but HM did not differ between INT and CSG. The SH was also greatest for MON (28 ± 1.11; INT: 23.6 ± 1.11; CSG: 19.7 ± 1.37 cm; *P <* 0.003). Forage nutrients (digestible energy and crude protein) were largely similar across treatments and met requirements of horses at maintenance. Horse GT was lower in MON (22.6 ± 3.77 min/sub-plot) than in INT (31.9 ± 3.79 min/sub-plot; *P* = 0.003) and there was a trend for lower GT in MON vs. CSG (29.9 ± 4.17 min/sub-plot: *P =* 0.07). These results indicate interseeding CRB would not effectively increase yields of traditional cool-season grass equine rotational grazing systems and would not supply similar levels of summer forage provided by monoculture establishment. Results of this study also suggest horses may prefer cool-season grass pasture forage over warm-season crabgrass.

## INTRODUCTION

Incorporating warm-season grasses has been suggested as a strategy for bridging the “summer slump” forage gap characteristic of traditional cool-season grass grazing systems in temperate regions of the United States (Moore et al., 2004; Guretzky et al., 2020; Ritz et al., 2020). Differences in photosynthetic mechanisms between these two grass types result in differences in seasonal growth patterns (Taiz and Zeiger, 2002). Cool-season grasses are most productive during cooler periods of the growing season (16–24 °C) in the late spring to early summer, with another secondary peak in the fall. Conversely, growth of warm-season grasses is most vigorous during hot, dry summer months (23–35 °C) (Fry and Huang, 2004; Jensen et al., 2014; DeBoer et al., 2017). Implementing an integrated warm- and cool-season grass rotational grazing approach can increase summer pasture yield (Moore et al., 2004; Guretzky et al., 2020; Ritz et al., 2020).

Warm-season grasses may be established in monoculture or interseeded into existing cool-season grass stands. Most studies that have evaluated integrated rotational grazing have utilized sequential grazing of distinct and separate pasture sections containing either cool-season or warm-season grasses (Moore et al., 2004; Hudson et al., 2010; Ritz et al., 2020). Monoculture establishment of warm-season grasses in separate pasture sections can prevent potential competition between grass types that could negatively impact establishment and depress yield of warm-season grasses or long-term persistence and production of established cool-season grasses (Reinbott and Blevins, 1995). Conversely, interseeding of warm-season grasses would improve land-use efficiency of available pasture areas (Guretzky et al., 2020). Furthermore, no-till monoculture establishment of warm-season grasses requires application of herbicide to eliminate existing forage, whereas warm-season grasses can be seeded into cool-season grass stubble following harvest or grazing. This reduction in labor and herbicide expense may be attractive to producers. However, research evaluating interseeding of warm-season grasses into cool-season grass pastures is limited comparatively (Clapham et al., 2011; Guretzky et al., 2020, 2021), and this practice has not been assessed under equine grazing.

Horses are considered selective or “patch” grazers, commonly overgrazing preferred grasses (Archer, 1973; Fleurance et al., 2001; Ménard et al., 2002). Overgrazing can have long-term impacts on pasture forage production, persistence, and botanical composition (Martinson et al., 2015; Weinert and Williams, 2018). Forage species, maturity, sward height, and nutrient composition have been identified as potential factors affecting horse forage preference (Allen et al., 2013; Martinson et al., 2015; Catalano et al., 2020). Due to seasonal growth patterns, differences in maturity and sward height between warm-season and cool-season grasses would be expected during summer months when warm-season grasses are most productive and cool-season grasses are semi-dormant. Additionally, there are characteristic differences in nutrient content in warm-season vs. cool-season grasses, with previous studies reporting higher fiber and lower nonstructural carbohydrate (NSC) and crude protein (CP) in warm-season vs. cool-season grasses (Moore et al., 2004; Jensen et al., 2014; DeBoer et al., 2017).

Many warm-season annual grasses typically utilized in cattle grazing systems may not be appropriate for horse pastures due to concerns about forage-related disorders (Teutsch, 2006; DeBoer et al., 2017). Improved forage varieties of crabgrass [*Digitaria sanguinalis* (L.) Scop.] may offer a viable summer grazing option as crabgrass does not contain prussic acid and accumulation of toxic levels of nitrates is rare (Teutsch et al., 2005, 2006; Weinert-Nelson et al., 2021). However, few studies have evaluated improved crabgrass varieties as horse pasture forages (Jaqueth et al., 2021; Weinert-Nelson et al., 2021), and no studies have evaluated the impact of establishment method (monoculture vs. interseeded) on crabgrass production and nutritive value or grazing horse preference. Therefore, the objective of this study was to determine the impact of establishment method on crabgrass pasture forage yield, nutritive value, and preference under equine grazing.

## MATERIALS AND METHODS

### Plot Establishment and Maintenance

This study was conducted in 2019 at the Ryders Lane Environmental Best Management Practices Demonstration Horse Farm (Rutgers, The State University of New Jersey; New Brunswick, New Jersey; geographic coordinates: 40°28ʹ9″ N, 74°25ʹ43″ W). Pasture soil was a silty clay loam comprised of FapA (Fallsingotn loams, 0%–2% slopes, Northern Coastal Plain), NknB (Nixon loam, 2%–5% slopes), and NkrA (Nixon moderately well-drained variant loam, 0%–2% slopes) (Weinert and Williams, 2018; Williams et al., 2020). Bi-annual soil tests are conducted at the study site, and lime and fertilizers were applied to adjust soil fertility to optimum ranges based on soil test results, with the most recent applications preceding initiation of grazing in 2019. Weather data from the New Brunswick station nearest to the site was obtained from the Historical Monthly Station Data portal of the Office of the New Jersey State Climatologist website (Rutgers New Jersey Weather Network 2020; https://www.njweather.org/data).

**Figure 1.**
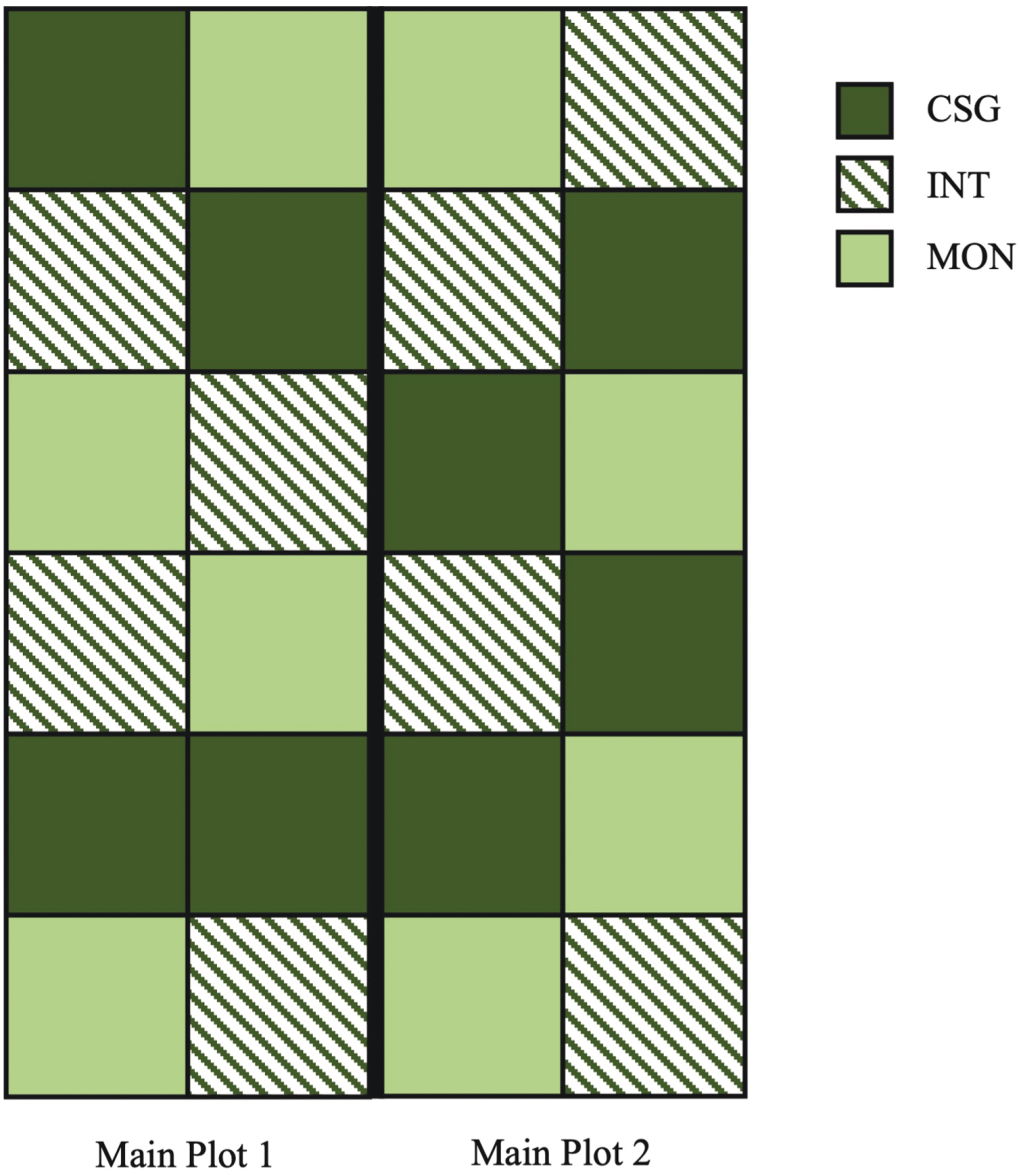
Diagram of pasture plots. Each main plot was divided into 12 subplots (6.1 m × 6.1 m), with four replicates of the three treatments: mixed cool-season grass only (CSG), *Quick-N-Big* crabgrass interseeded into existing mixed cool-season grass (INT), and *Quick-N-Big* crabgrass established as a monoculture (MON). Main plots were adjacent with one shared fence line.

Impacts of establishment method were evaluated using a randomized complete block design (Grev et al., 2017). Individual sub-plot dimensions were 6.1 m × 6.1 m. Two main plots were established. Main plots were adjacent and had one shared fence line. Each main plot contained four replicates of three treatments (total of 12 subplots per main plot): (1) mixed cool-season grass (CSG); (2) *Quick-N-Big* crabgrass (CRB; Dalrymple Farms, Thomas, OK) interseeded into existing cool-season grass (INT); and (3) CRB established as a monoculture (MON). A diagram of the plot design utilized in this study is shown in Fig. 1. The cool-season grass mix contained *Inavale* orchardgrass (OG) [*Dactylis glomerata* (L.)], endophyte-free *Tower* tall fescue (TF) [*Lolium arundinaceum* (Schreb.) Darbysh.], and *Argyle* Kentucky bluegrass (KB) [*Poa pratensis* (L.)] (DLF Pickseed, Halsey, OR). These grasses were planted across the full study area in a 24-16-16 mix (total seeding rate = 56 kg/ha) in the fall of 2017 using a no-till drill after glyphosate was applied to eliminate existing forage. Seeding mixtures and rates are summarized in Table 1. Pasture area used for this study was not grazed in 2018 and was managed using minimal mowing to control weed growth. In June 2019, the cool-season grass was mowed and glyphosate was applied to kill the cool-season grasses in MON subplots. On June 3, crabgrass was planted at 13 kg/ha in MON and 7 kg/ha in INT by no-till drilling. A 0.3-m buffer zone was maintained around all subplots through spot application of glyphosate. Following initial germination of CRB, N fertilizer was applied to all subplots at a rate of 33.6 kg/ha. Subsequent applications were conducted following the first and second grazing events. For weed control, one application of 2,4-D (2.33 L/ha) was required prior to the second grazing event.

**Table 1.** Seeding mixtures and seeding rates of pasture forages.

Treatment	Grass species	Seeding rate, kg/ha
CSG^1^	*Inavale* orchardgrass [*Dactylis glomerata* (L.)]	26
	*Tower* tall fescue [*Lolium arundinaceum* (Schreb.) Darbysh.]	16
	*Argyle* Kentucky bluegrass [*Poa pratensis* (L.)]	16
INT^2^	*Inavale* orchardgrass [*Dactylis glomerata* (L.)]	26
	*Tower* tall fescue [*Lolium arundinaceum* (Schreb.) Darbysh.]	16
	*Argyle* Kentucky bluegrass [*Poa pratensis* (L.)]	16
	*Quick-N-Big* crabgrass [*Digitaria sanguinalis* (L.) Scop.]	7
MON^3^	*Quick-N-Big* crabgrass [*Digitaria sanguinalis* (L.) Scop.]	13

Cool-season grasses (CSG) were established prior to the study period in the fall of 2017.

Crabgrass was interseeded (INT) into existing cool-season grass (mowed prior to planting) using a no-till drill in June 2019.

Crabgrass was established in monoculture (MON) using a no-till drill in June 2019 following application of glyphosate to kill existing forage.

### Horses and Grazing Events

Use of animals in this study was approved by the Rutgers University Institutional Animal Care and Use Committee protocol #PROTO201800013. Three adult Standardbred mares (552 ± 31 kg body weight [BW]; 17 ± 1 yr) were utilized in this study. All horses (*n* = 3) grazed each main plot for an 8-hr grazing event (2 days of grazing, one day in each main plot per grazing event). Grazing events were repeated three times across the grazing season on Jul 28/29 (GRAZE 1), Aug 29/30 (GRAZE 2); and Oct 1/2 (GRAZE 3). Horses had access to all replicates within the main plot during the grazing event (horses grazed the entire main plot simultaneously). Horses also had ad libitum access to water, which was placed in the center of the main plot within the buffer zone around individual-subplots. When not grazing plots for the current study, horses were maintained in an integrated rotational grazing system containing the same mixed cool-season grass and monoculture *Quick-N-Big* crabgrass established in separate pasture sections. Prior to the first grazing event horses had completed rotations in both cool-season grass and crabgrass sections within the integrated system. Thus, *Quick-N-Big* was not a novel pasture forage for horses used in this establishment method study. Twenty-four hours before each grazing event, horses were confined to a dry lot within the integrated rotational system and fed a mixed cool-season grass hay. Horses were moved to the main plot and grazing events initiated at 0800 h (completion at 1600 h).

### Pasture and Preference Measurements

Pasture measures were collected prior to each grazing event (0700–0800 h) to assess yield, persistence, maturity, and nutritive value. Herbage mass (HM) was determined by hand-clipping forage (to 7.6 cm to represent minimum recommended grazing height) within a 0.25 m × 0.25 m PVC quadrat placed randomly at three sites in each subplot. Forage was collected in a paper bag, dried at 60 °C in a Thelco (Precision Scientific, Chicago, IL) drying oven to remove moisture content. The dry-matter (DM) weight was then used to estimate HM (Williams et al., 2020). Sward height (SH) was measured by dropping a styrofoam plate down a meter stick, with height recorded where the plate rested on the pasture forage (Burk et al., 2011; Williams et al., 2020). Maturity was assessed using index scoring adapted from previously described methods (Moore et al., 1991). For the current study, a maturity index score (MIS) of 1–5 was assigned to each plant evaluated based on growth stages, with 1 = emergence, 2 = presence of leaves, 3 = stem elongation, 4 = boot stage, and 5 = presence of seed heads. Species composition was assessed using the step-point method (Evans and Love, 1957; Kenny et al., 2018). Individual observations were classified as either TF, KB, OG, CRB, or O (for any observation other than a planted grass species). For SH, maturity, and species composition, 10 measures were collected per subplot by traversing the subplot in a random zig-zag pattern, stopping every 10 paces to collect measurements. Hand-clipped forage samples were collected for each of the subplots to evaluate chemical composition. After drying for at least 36 hr in a Thelco oven (Precision Scientific, Chicago, IL) at 60 °C to determine DM, samples were ground to 1 mm using a Wiley Mill. Samples were pooled by treatment within each main plot with an equal sample weight for each replicate contributed to the pooled treatment samples. Pooled samples were submitted to Equi-Analytical Laboratories (Ithaca, NY) to be analyzed by wet chemistry.

Methods utilized for analysis of chemical composition by Equi-Analytical include the following: Crude protein (CP) was analyzed with a Leco FP-528 Nitrogen/Protein Analyzer (Leco Corporation, St. Joseph, MI) according to protocols established by the Association of Official Analytical Chemists (AOAC 990.03). Acid detergent fiber (ADF) and neutral detergent fiber (NDF) were analyzed with an ANKOM A200 Digestion Unit (ANKOM Technology, Macedon, NY) (ANKOM Technology Method 5 and Method 6, respectively). Digestion solutions for ADF were as specified by AOAC 973.18 and for NDF by Van Soest et al. (1991). Water-soluble carbohydrates (WSC) and ethanol soluble carbohydrates (ESC) were extracted and analyzed following methods detailed by Hall et al. (1999). Colorimetric analyses of WSC and ESC concentrations were conducted using a Thermo Scientific Genesys 10S Spectrophotometer (Thermo Fisher Scientific, Inc., Waltham, MA). Starch was determined using a YSI 2700 SELECT Biochemistry Analyzer (YSI Incorporated Life Sciences, Yellow Springs, OH). Nonstructural carbohydrates were calculated as WSC + starch. Calcium and phosphorus were analyzed using a Thermo iCAP 6300 Inductively Coupled Plasma (ICP) Radial Spectrometer (Thermo Fisher Scientific, Inc., Waltham, MA) following microwave digestion with a CEM Microwave Accelerated Reaction System (MARS) with MarsXpress Temperature Control using calibrated Xpress Teflon PFA vessels with Kevlar/fiberglass insulating sleeves (CEM, Matthews, NC). The method utilized for microwave digestion was based upon CEM Application Notes for Acid Digestion on the following matrices—Feed Grain, Alfalfa, Corn Flour, Milk Powder, Soybean Meal, Flour, Hair, Potato Chips, Wheat Crackers, Peanut Butter, Urine, Dog Feces, Wine. Digestible energy was estimated with the equation: DE (kcal/kg DM) = 2118 + 12.18 (CP %) − 9.37(ADF %) − 3.83(hemicellulose %) + 47.18(fat %) + 20.35(NSC %) − 26.3% ash) (Pagan, 1998).

### Preference Assessment

Horse preference was assessed by percentage forage removal based on pre- and post-grazing SH measurements and time spent grazing in each subplot. Horses were observed during grazing by two trained observers. Observers were stationed close enough to grazing plots so that buffer zone boundaries for each section could be seen without obstruction, but not so close that observer proximity would impact horse grazing activity. Observers were provided with a plot map in which individual subplots were assigned a number. Observations were collected using a 5-min scan sampling technique (Werner et al., 2018; Weinert et al., 2020), in which observers recorded the section number individual horses were grazing in at 5-min intervals. If a horse was not grazing at the time an observation was collected, no subplot number was recorded and NG (not grazing) was reported as the observation for that time. Observational data were averaged between the two observers and was used to determine the percentage of time spent grazing each treatment (GT). After completion of each grazing event, the postgrazing SH was measured using the method described for pre-grazing SH to calculate percent forage removal.

### Statistical Analysis

Based on Step-Point observational data, four CSG subplots contained >10% CRB (likely attributable to drift during planting) at the time of at least one grazing event, and these subplots were removed from the dataset. Mean HM, pre- and post-grazing SH, percentage G, and percent removal were then analyzed using a mixed model ANOVA in R (v. 4.0.2) with treatment, grazing event, their interactions as fixed factors, and replicate nested within main plot considered as the random effect. For total HM, the model included treatment as a fixed factor and replicate nested within main plot as the random factor. For forage chemical composition, the model included treatment and grazing event as fixed effects with main plot as the random factor. The mean GT per subplot was also assessed using mixed model ANOVA, with treatment, grazing event, and their interactions as fixed factors; horse and replicate nested within main plot were set as random factors. Means were separated using Tukey’s method. Normality of residuals was assessed using a Shapiro–Wilk test. The species composition frequency counts of cool-season grasses (KB, O, TF) within CSG and INT were analyzed using Fisher’s exact test as expected counts from Pearson’s Chi Square Test of Association were <5 in >20% of cells and/or cells had an expected count of <1. Relationships between preference metrics and forage characteristics were assessed using Pearson correlations. Results were considered significant at *P ≤* 0.05; trends were considered at *P ≤* 0.10. Data are presented as means ± SEM.

## RESULTS AND DISCUSSION

### Forage Yield and Persistence

**Figure 2.**
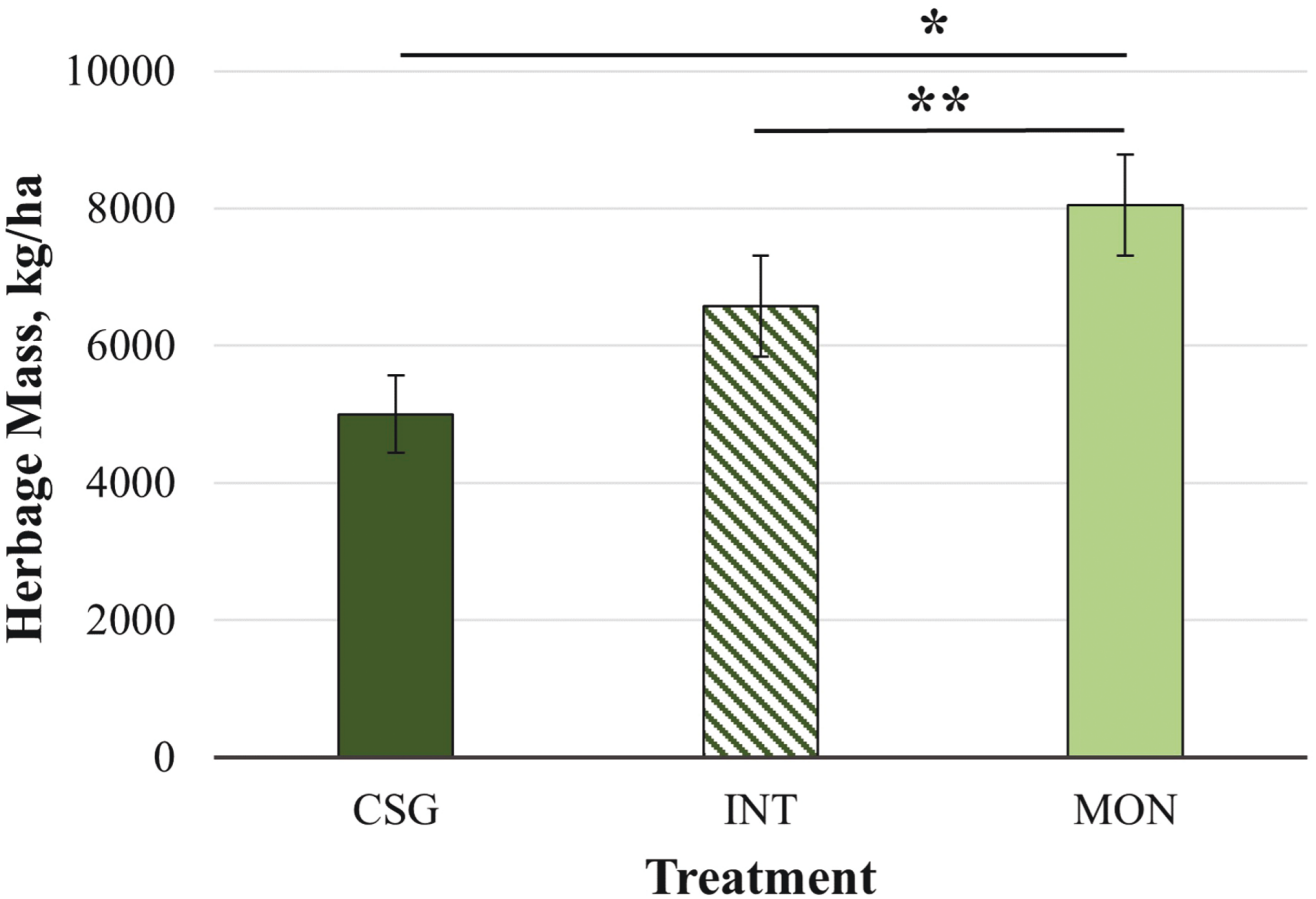
Total herbage mass (kg/ha) by treatment: mixed cool-season grass only (CSG), *Quick-N-Big* crabgrass interseeded into existing mixed cool-season grass (INT), and *Quick-N-Big* crabgrass established as a monoculture (MON). Cool-season grass species in pasture mix included *Argyle* Kentucky bluegrass, *Inavale* orchardgrass, and *Tower* tall fescue. A single asterisk indicates differences between treatments at *P ≤* 0.05. Double asterisks indicate a trend for differences *P ≤* 0.10. Data are presented as the means ± SEM.

**Figure 3.**
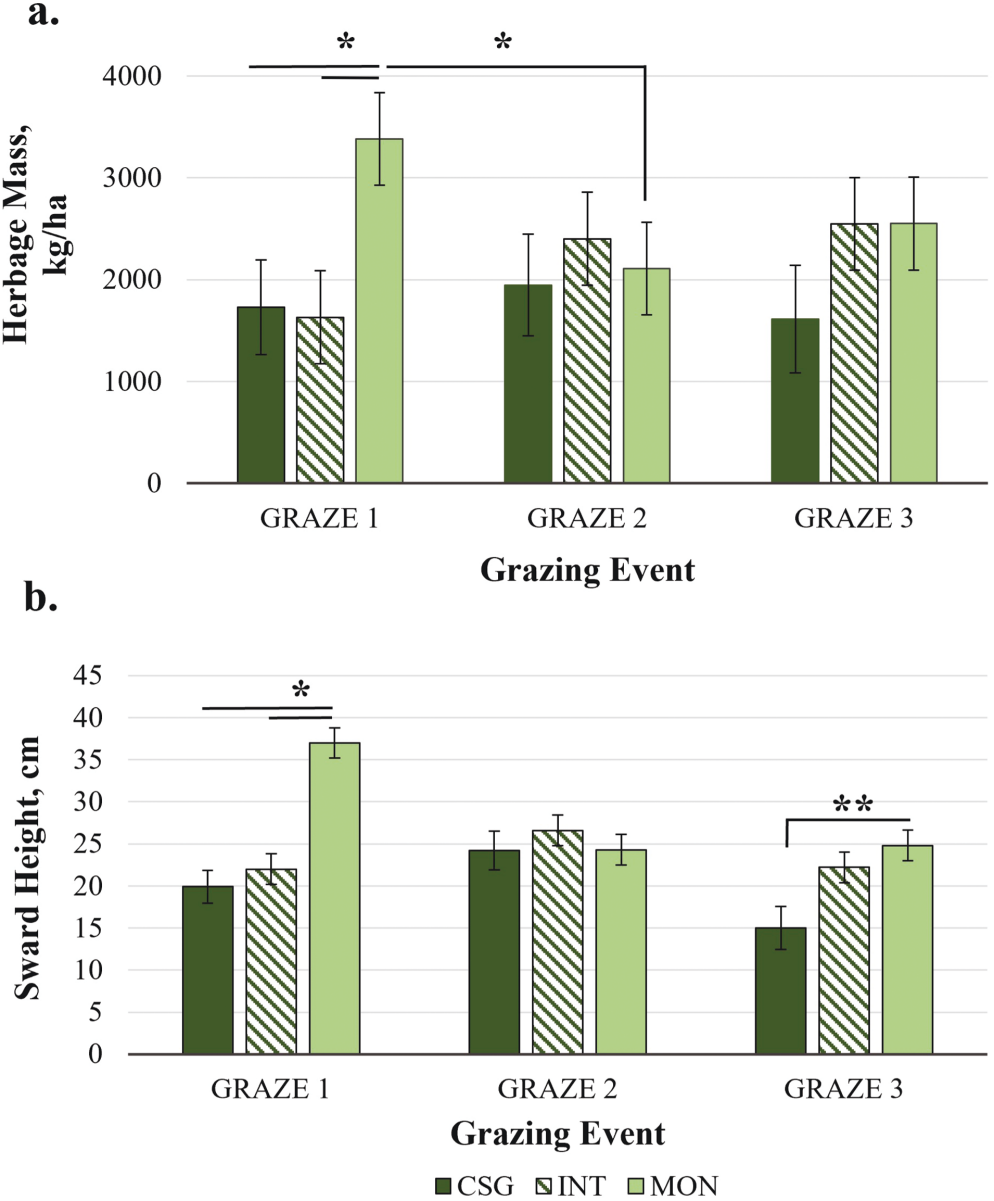
Pasture yield by treatment [mixed cool-season grass only (CSG), *Quick-N-Big* crabgrass interseeded into existing mixed cool-season grass (INT), and *Quick-N-Big* crabgrass established as a monoculture (MON)] across three grazing events in 2019: Jul 29/30, 2019 (GRAZE 1), Aug 29/30 (GRAZE 2), Oct 1/2 (GRAZE 3) including pre-grazing (a) herbage mass (HM; kg/ha)) and (b) sward height (SH; cm). Cool-season grass species in pasture mix included *Argyle* Kentucky bluegrass, *Inavale* orchardgrass, and *Tower* tall fescue. A single asterisk indicates differences between treatments within grazing events at *P ≤* 0.05. Double asterisks indicate a trend for differences *P ≤* 0.10. Data are presented as the means ± SEM.

Monoculture establishment of CRB resulted in the greatest forage yield. Total HM (summed yield across all three grazing events) differed by treatment (*P =* 0.003), with total HM greater in MON (8043 ± 1220 kg/ha) than CSG (5001 ± 1308 kg/ha; *P =* 0.003) and a trend for greater total HM in MON vs. INT (6582 ± 1220 kg/ha; *P =* 0.06; Fig. 2). There was also a treatment by grazing event interaction for both mean HM and SH (*P <* 0.002; Fig. 3a–b). Mean HM and SH in MON (HM: 3383 ± 456 kg/ha; SH: 37.0 ± 1.82 cm) were greater than in CSG (HM: 1730 ± 467 kg/ha; SH: 19.9 ± 1.95 cm) or INT (HM: 1631 ± 456 kg/ha: SH: 22.0 ± 1.82 cm) during GRAZE 1 (*P <* 0.002). There were, however, no differences by treatment in either the second or third grazing event for either HM or SH. Mean HM and SH in MON for GRAZE 1 were greater compared to GRAZE 2 in MON (HM: 2110 ± 456 kg/ha; SH: 24.3 ± 1.82 cm; *P <* 0.02). Mean SH for MON for GRAZE 1 was also greater than for GRAZE 3 (SH: 24.8 ± 1.82 cm; *P =* 0.004).

Total HM in the CSG treatment were comparable to mixed cool-season grass horse pasture yields in Minnesota (6100–7082 kg/ha) reported by Martinson et al. (2015), but lower than yields reported by Allen et al. (2012) for monoculture cool-season grasses (8,600–11,100 kg/ha). Total CSG HM in the current study were also above previous yields reported for CSG rotational horse pastures (4,223 kg/ha) at the same study site in 2019 (Weinert-Nelson et al., 2021). However, the current study did not assess early or late-season CSG yields outside of the CRB growing period. Thus, it is likely that full-season yields of CSG would have fallen within the higher range of yields reported by Allen et al. (2012). The HM by grazing event for CSG was similar to per-rotation averages (1500–3160 kg/ha) for other cool-season rotational grazing systems at the same research site in previous years (Williams et al., 2020). Total HM in MON was also greater than monoculture *Quick-N-Big* crabgrass sections (5,848 kg/ha) of a crabgrass and cool-season grass integrated rotational equine grazing system managed in separate pasture fields at the study site in 2019 (Weinert-Nelson et al., 2021). The MON HM was within yield ranges reported for *Quick-N-Big* crabgrass in Tennessee (1,888–7,501 kg/ha; Gelley et al., 2016) and Oklahoma (5,459–15,680 kg/ha; Bouton et al., 2019). The HM and SH for MON were greater in the GRAZE 1 vs. GRAZE 2. Thus, while total yield was greatest in MON, yield differences were primarily attributable to the first grazing event when MON was highly productive. Other improved crabgrass varieties such as *Red River* and *Impact* do reach peak yield later in the growing season than *Quick-N-Big* (Bouton et al., 2019). Therefore, a crabgrass mixture that includes these later-yielding varieties may improve yield performance in later months of the grazing season.

Results of the current study suggest that establishment of CRB by interseeding into existing cool-season pasture grasses will not produce similar increases in summer forage yields as monoculture establishment. Total HM in MON was 61% greater than in CSG, while total HM in INT was only 32% greater than CSG. However, if implemented in a full-pasture setting, horses would have access to interseeded sections during early grazing months when cool-season grasses are productive. If established in monoculture, crabgrass sections would remain nonproductive (and thus unavailable for grazing) during this period. Similarly, in the later fall months as cooler temperatures return, cool-season grasses still growing in interseeded sections would provide additional pasture forage, while crabgrass production would be declining, leaving monoculture sections less productive in late-season grazing. Thus, the production advantage for MON vs. INT during summer and early fall may be partially offset by yield losses in early- and late-season grazing.

Weinert-Nelson et al. (2021) suggested that by lowering grazing pressure during early grazing periods, interseeding could potentially improve late-season production of cool-season pasture sections in crabgrass integrated equine rotational grazing systems. However, results of the current study indicate that this strategy would sacrifice summer production in comparison to an integrated system with monoculture establishment, without improving production over traditional cool-season grass pastures during this period. A 32% increase in pasture production for INT vs. CSG is not negligible, but lack of statistical significance suggests limited value for increasing summer pasture yield over cool-season pastures. Additionally, while cool-season grass in interseeded pasture sections could be grazed in early months of the grazing season, grazing of these sections would need to be restricted following planting to allow time for CRB to establish, which could decrease grazing days and overall forage available for grazing, further narrowing any production advantage for interseeding vs. traditional cool-season pasture systems.

It should be noted that in the current study, INT was planted with CRB at half the rate of MON. This was reflected in the percentage of CRB in INT vs. MON (33% and 63%, respectively). Increasing the CRB planting rate for interseeding would likely increase the proportion of CRB, and this could lead to higher summer yields. Alternatively, the method of forage removal in preparing for CRB seeding might also have impacted production in INT. Guidance from the producer of *Quick-N-Big* crabgrass seed indicates that more robust establishment and yield of interseeded crabgrass is expected when utilizing grazing vs. mechanical removal of existing cool-season pasture grasses prior to planting (Dalrymple Farm, 1998). Small-plot pasture areas used in the current study could not be grazed prior to planting CRB, and cool-season forage was removed by mowing. In a practical management setting, cool-season grasses would be grazed during spring and early summer prior to interseeding, which could improve yield. However, the percentage CRB in MON in the current study was well below the 82%–87% reported for monoculture CRB sections in a full integrated rotational grazing system managed at the study site in 2019 (Weinert-Nelson et al., 2021). A greater proportion of CRB in MON such as the percentage documented in full pasture sections would have potentially increased yields in MON and could negate any potential gains from increasing CRB planting rates for interseeding. Additionally, higher CRB seeding rates may potentially have negative long-term effects on persistence of planted cool-season grasses, as increases in the proportion of crabgrasses over time have been documented (Coffey et al., 2005).

**Figure 4.**
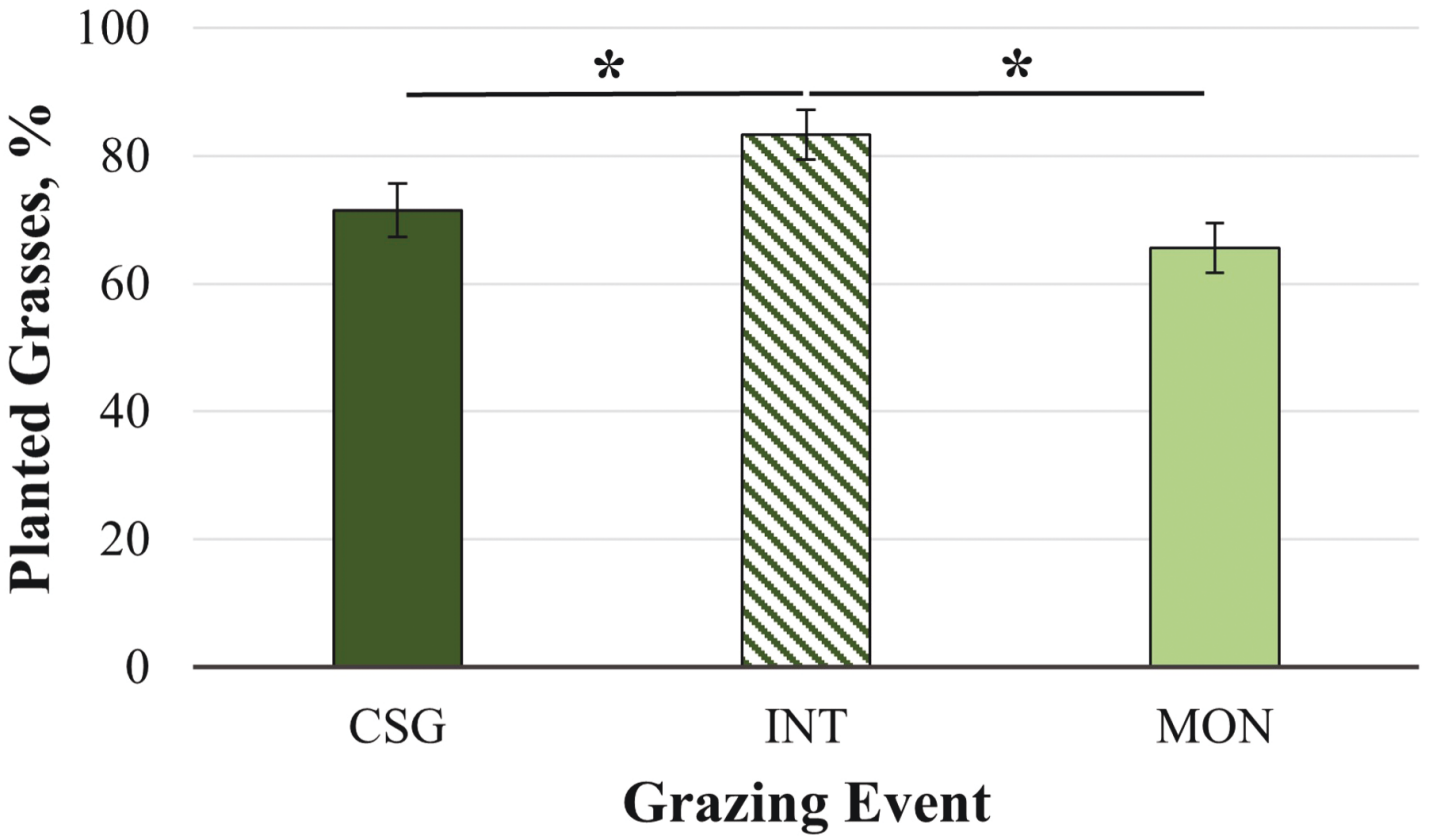
Percentage of planted grasses (G; %) by treatment: mixed cool-season grass only (CSG), *Quick-N-Big* crabgrass interseeded into existing mixed cool-season grass (INT), and *Quick-N-Big* crabgrass established as a monoculture (MON). Cool-season grass species in pasture mix included *Argyle* Kentucky bluegrass, *Inavale* orchardgrass, and *Tower* tall fescue. A single asterisk indicates differences between treatments at *P ≤* 0.05. Data are presented as the means ± SEM.

Multiyear studies would be beneficial in assessing species persistence of pasture grasses across treatments. In the current study, the percentage of G was greatest in INT (83.1 ± 3.91; MON: 65.5 ± 3.91; CSG: 71.5 ± 4.59%; *P <* 0.04; Fig. 4). This suggests that interseeding decreased the occurrence of weeds and instances of other observations such as bare ground. The percentage G did not differ by grazing event and there was not a significant treatment by grazing event interaction. Similarly, the distribution of planted cool-season grasses (KB, OG, TF) did not differ across treatments overall (Table 2) or for any of the grazing events. However, this study only evaluated changes over time across three months of one season. Management impacts on pasture vegetative cover and forage/species composition are more likely to emerge over a longer time-frame (years rather than months) (Weinert and Williams, 2018; Guretzky et al., 2020; Williams et al., 2020). Results of this study indicate that interseeding warm-season annual grasses such as crabgrass may represent a potential strategy for suppressing weed growth in cool-season grass pastures. Overgrazing, reduction of vegetative cover, and subsequent weed invasion is a challenge that is commonly encountered in horse pasture management (Weinert and Williams, 2018; Williams et al., 2020). Thus, additional research is needed to determine the utility of interseeding warm-season grasses for mitigating detrimental effects of overgrazing.

**Table 2.** Species composition (%)^1^ of planted grass species *Argyle* Kentucky bluegrass (KB), *Inavale* orchardgrass (OG), and *Tower* tall fescue (TF) in treatments containing mixed cool-season grass: cool-season grass only (CSG) and *Quick-N-Big* crabgrass interseeded into existing cool-season grass (INT).

Grass	Treatment^2^	
	CSG	INT
KB, %	3	4
OG, %	47	44
TF, %	54	68

Percentages are shown as means across three grazing events in July, August, and October 2019.

There were no differences in the distribution of grass species by treatment (analyzed by Fisher’s Exact Test).

A prior warm-season grass interseeding study by Guretzky et al. (2020) also found varying effects of interseeding on pasture yield across multiple study sites, years, and environments (humid vs. semi-arid). This is similar to findings of previous integrated grazing studies that have utilized monoculture warm-season grass sections, which have attributed differences between years to environmental conditions (Moore et al., 2004; Tracy et al., 2010; Ritz et al., 2020). The current study collected data in one year (2019) at one study location (New Brunswick, NJ). Expanded data collection in multiple years in multiple regional sites would be necessary to provide more robust determinations on the yield impacts of interseeding CRB into existing cool-season grass horse pastures, especially as average temperatures were above historical averages in July and September 2019 (weather data shown in Table 3). Total monthly precipitation was also above average for June and July, but well below average for September. Multiple-year studies would be particularly valuable for evaluation of crabgrass grazing systems and management practices. While crabgrass is a warm-season annual, if managed properly at the end of the grazing season, crabgrass can re-seed itself providing continued forage production in the subsequent grazing season.

**Table 3.** Weather data from 2019 grazing season. Data shown include monthly average temperature and total precipitation from 2019 as well as historical averages^1^.

Date	Average temperature, °C			Total precipitation, cm
	2019	Historical average	2019	Historical average
June	21.3	21.0	13.8	9.9
Jul	26.7	23.8	16.0	12.5
Aug	23.8	22.8	11.6	11.9
Sep	22.7	19.2	3.9	10.3

Weather data were obtained for the New Brunswick Station through the Office of the New Jersey State Climatologist website (Rutgers New Jersey Weather Network 2020; https://www.njweather.org/data).

### Forage Quality and Nutritive Value

Forage NSC, water-soluble carbohydrates (WSC), and ethanol-soluble carbohydrates (ESC) did not differ by treatment. The NSC concentrations did differ by grazing event, with NSC in GRAZE 3 (8.28 ± 0.63%) greater than for GRAZE 1 (4.72 ± 0.63%; *P =* 0.01). The WSC and ESC concentrations did not differ by grazing event; however, and there were no significant treatment by grazing event interactions for NSC, WSC, or ESC (Table 4). This increase in NSC in later grazing events was thus attributable to increases in starch, which was greatest in GRAZE 3 (4.05 ± 0.35%) and was also greater in GRAZE 2 (1.93 ± 0.35%) than in GRAZE 1 (0.55 ± 0.35%; *P ≤* 0.05). Starch also differed by treatment and was lower in CSG (1.28 ± 0.35%) than MON (2.72 ± 0.35%; *P =* 0.04) or INT (2.53 ± 0.35%; *P =* 0.08). There was also a trend for a treatment by grazing event interaction for starch, with starch in CSG lower than in MON for GRAZE 3 (*P =* 0.06; Table 4). Other pairwise differences for the interaction term were all by grazing event within treatments, with starch lower in both INT and MON for GRAZE 1 vs. GRAZE 3 (*P <* 0.04). Starch was also lower in MON during GRAZE 2 compared to GRAZE 3 (*P =* 0.03).

**Table 4.** Nutrient composition^1^ by treatment [cool-season grass only (CSG) and crabgrass either interseeded into existing cool-season grass (INT) or established as monoculture (MON)] across three grazing events: GRAZE 1 (Jul 28/29, 2019), GRAZE 2 (Aug 29/30, 2019), and GRAZE 3 (Oct 1/2, 2019). Cool-season grass species in pasture mix included *Argyle* Kentucky bluegrass, *Inavale* orchardgrass, and *Tower* tall fescue.

Nutrient^2^	Treatment					*P*-Value		
	CSG	INT	MON	Mean	SEM^3^	Treatment	Grazing Event	Treatment × Grazing Event
Dry matter, %	22.8	24.4	22.2		3.13	NS^4^	NS	NS
GRAZE 1	20.5	26.4	19.5	22.1	3.51			
GRAZE 2	20.4	24.4	21.6	22.2	3.51			
GRAZE 3	27.7	22.5	25.5	25.1	3.51			
Digestible energy, Mcal/kg^5^	2.15	2.14	2.15		0.02	NS	.010	.024
GRAZE 1	2.08	2.08	2.20	2.12^A^	0.03			
GRAZE 2	2.13	2.15	2.05	2.11^A^	0.03			
GRAZE 3	2.23	2.18	2.18	2.20^B^	0.03			
Crude protein, %	27.0^c^	24.5^b^	21.8^a^		0.52	<0.001	NS	0.033
GRAZE 1	25.8	25.6	24.8	25.4	0.91			
GRAZE 2	26.9^b^	23.9^ab^	20.4^a^	23.7	0.91			
GRAZE 3	28.4^by^	24.1^x^	20.2^a^	24.2	0.91			
Acid detergent fiber, %	31.6	31.3	32.1		0.28	NS	0.006	<0.001
GRAZE 1	34.0^b^	32.5^y^	30.0^ax^	32.1^B^	0.48			
GRAZE 2	31.4^ab^	31.1^a^	33.9^b^	32.1^B^	0.48			
GRAZE 3	29.3^a^	30.2^ab^	32.5^b^	30.7^A^	0.48			
Neutral detergent fiber, %	55.4	56.6	57.1		0.662	NS	0.012	0.016
GRAZE 1	58.0	58.4	54.4	56.9^BY^	1.15			
GRAZE 2	56.2	56.2	61.1	57.9^B^	1.15			
GRAZE 3	52.0	55.2	55.9	54.4^AX^	1.15			
Water-soluble carbohydrate, %	4.32	4.18	4.15		0.21	NS	NS	NS
GRAZE 1	4.15	4.20	4.15	2.17	0.37			
GRAZE 2	4.55	3.85	4.35	4.25	0.37			
GRAZE 3	4.25	4.50	3.95	4.23	0.37			
Ethanol soluble carbohydrate, %	3.07	3.13	3.03		0.20	NS	NS	NS
GRAZE 1	3.50	3.15	2.65	3.10	0.35			
GRAZE 2	2.75	2.85	3.10	2.90	0.35			
GRAZE 3	2.95	3.440	3.35	3.23	0.35			
Starch (%)	1.28^ax^	2.53^y^	2.72^b^		0.35	0.033	<0.001	0.096
GRAZE 1	0.60	0.50	0.55	0.55^A^	0.60			
GRAZE 2	0.90	2.95	1.95	1.93^B^	0.60			
GRAZE 3	2.35^x^	4.15^xy^	5.65^y^	4.05^C^	0.60			
Nonstructural Carbohydrate, %^6^	5.60	7.25	6.87		0.63	NS	0.010	NS
GRAZE 1	4.75	4.70	4.70	4.72^A^	1.09			
GRAZE 2	5.45	8.40	6.30	6.72^AB^	1.09			
GRAZE 3	6.60	8.65	9.60	8.28^B^	1.09			

Nutrient composition of forage samples was determined by wet chemistry (Equi-Analytical Laboratories, Ithaca, NY). Concentrations, except dry matter, are reported on a dry-matter basis.

Data are presented as the means.

SEM, standard error of the mean

NS, main effect or interaction was not significant nor was there a trend (*P* > 0.10).

Digestible energy was estimated with the equation: DE (kcal/kg DM) = 2,118 + 12.18 (CP %) − 9.37 (ADF %) − 3.83 (hemicellulose %) + 47.18 (fat %) + 20.35 (NSC %) − 26.3% ash) (Pagan, 1998).

Nonstructural carbohydrates were calculated as the sum of water-soluble carbohydrates and starch.

Indicates significant difference within rows (*P ≤* 0.05).

Indicates a trend for a difference within rows (*P ≤* 0.10).

Indicates significant difference within column (*P ≤* 0.05).

Indicates a trend for a difference within column (*P ≤* 0.10).

Forage digestible energy (DE), acid detergent fiber (ADF), and neutral detergent fiber (NDF) did not differ by treatment. These nutrient fractions did differ by grazing event (*P ≤* 0.01) and there were significant treatment by grazing event interactions for these nutrients (*P <* 0.03; Table 4). However, the only pairwise difference for the DE interaction term was a trend for lower DE in CSG for GRAZE 1 than GRAZE 3; *P =* 0.10). For GRAZE 1, ADF was greater in CSG than MON (*P =* 0.006; Table 4). For GRAZE 2, however, ADF was greater in MON compared to INT (*P =* 0.05), and there was a trend for greater ADF in MON than CSG (*P =* 0.08). For GRAZE 3, ADF again was greater in MON than CSG (*P =* 0.02), but there was no difference between MON and INT or INT and CSG. Within treatments, ADF was greater in CSG for GRAZE 1 than GRAZE 3 (*P =* 0.002), and there was a trend for greater CSG ADF in GRAZE 1 vs. GRAZE 2 (*P =* 0.06). Conversely, the ADF in MON was lower in GRAZE 1 compared to GRAZE 2 (*P =* 0.007). Pairwise comparisons of the NDF interaction terms did not reveal any differences between treatments within grazing events, but similar to ADF, the NDF concentrations in MON were lower for GRAZE 1 vs. GRAZE 2 (*P =* 0.04), and in CSG, there was a trend for greater NDF for GRAZE 1 than GRAZE 3 (*P =* 0.08).

In contrast to other nutrients, CP did differ by treatment (*P =* 0.0002). Forage CP was not only greatest in CSG but also was greater in INT than in MON (*P <* 0.02). There was also a trend for differences in CP by grazing event (*P =* 0.010) and a significant treatment by grazing event interaction (*P =* 0.02). For GRAZE 2 and GRAZE 3, CP was greater in CSG vs. MON (*P <* 0.009; Table 4). Within MON, there was a trend for greater CP concentrations in both GRAZE 2 and GRAZE 3 compared to GRAZE 1 (*P <* 0.08).

The above-noted differences in forage nutrient composition are likely related to maturity of forages within treatments across the grazing events. Maturity varied by both treatment and grazing event, and there was a significant treatment by grazing event interaction (*P <* 0.01; Table 5). The MIS was greater in MON (4.33 ± 0.07) than in either CSG (3.31 ± 0.08) or INT (3.60 ± 0.07) and was also greater for INT vs. MON (*P <* 0.03). Across all treatments, MIS was lower at the time of GRAZE 1 (3.36 ± 0.07) compared to GRAZE 2 (3.92 ± 0.07) or GRAZE 3 (3.97 ± 0.07; *P <* 0.0001). These changes in maturity mirror increases in starch and fiber and decreases in CP across grazing events.

**Table 5.** Maturity Index Score^1^ of treatments [crabgrass established as monoculture (MON) or interseeded into existing cool-season grass (INT), and cool-season grass only (CSG)] across three grazing events: GRAZE 1 (Jul 28/29, 2019), GRAZE 2 (Aug 29/30, 2019), and GRAZE 3 (Oct 1/2, 2019). Cool-season grass species in pasture mix included *Argyle* Kentucky bluegrass, *Inavale* orchardgrass, and *Tower* tall fescue.

Grazing event	Treatment^2^				*P*-Value		
	CSG	INT	MON	MEAN	Treatment	Grazing event	Treatment × Grazing Event
GRAZE 1	3.19 ± 0.13^a^	3.10 ± 0.12^b^	3.79 ± 0.12^b^	3.36 ± 0.07^A^	<0.0001	<0.0001	0.04
GRAZE 2	3.50 ± 0.14^a^	3.88 ± 0.12^b^	4.40 ± 0.12^b^	3.92 ± 0.07^B^			
GRAZE 3	3.25 ± 0.17^a^	3.84 ± 0.12^b^	4.81 ± 0.12^b^	3.97 ± 0.08^B^			
MEAN	3.43 ± 0.08^a^	3.60 ± 0.07^a^	4.33 ± 0.07^b^				

Maturity was assessed using index scoring adapted from Moore et al., (1991) such that scores of 1-5 were assigned for the following stages: 1 = emergence; 2 = leaf; 3 = stem; 4 = boot; 5 = seed head.

Maturity varied by both treatment and grazing event, and there was a significant treatment by grazing event interaction (*P <* 0.01). Data are presented as the means ± SEM.

Indicates significant difference within rows (*P ≤* 0.05).

Indicates significant difference within column (*P* ≤ 0.05).

These results indicate that grazing both MON and INT would meet (and likely exceed) nutritional requirements of horses at maintenance. The average DE values were 2.14 Mcal/kg for each of these treatments. At an intake rate of 2.5% BW DM, a 500-kg horse would thus exceed daily energy and protein requirements (NRC, 2007). Furthermore, NSC concentrations for all treatments remained below 10%, the critical threshold for horses requiring limited dietary NSC intake (Frank et al., 2010). Warm-season grasses have been suggested as low-NSC alternatives to traditional cool-season grass pasture forage (Bott et al., 2013; DeBoer et al., 2017; Ghajar et al., 2021), despite prior reports of low NSC in both warm- and cool-season pasture grasses during summer and early fall months (when both forage types would be available for grazing) (Jensen et al., 2014; DeBoer et al., 2018; Weinert-Nelson et al., 2021). However, numerous factors can influence production and accumulation of NSC in growing plants (Taiz and Zeiger, 2002). Summer and early fall NSC levels in excess of 10% have been documented, and higher NSC is more common if the pasture contains perennial ryegrass [*Lolium perenne* (L.)] (Allen et al., 2013; Grev et al., 2017; Catalano et al., 2020). Therefore, results of this study support previous findings that *Quick-N-Big* crabgrass may not provide benefit for limiting NSC intake (Weinert-Nelson et al., 2021). However, under different growing, management, and environmental conditions that produce higher NSC in cool-season grasses, *Quick-N-Big* could provide a lower-NSC alternative. Additionally, it should be noted that pasture samples in the current study were collected by 0800 h prior to the start of each grazing event. Diurnal fluctuations in pasture forage NSC have been widely reported, with highest concentrations present in the late afternoon and early evening (Kagan et al., 2011, 2018), and Weinert-Nelson et al. (2022) reported greater increases in NSC for cool-season grasses in comparison to warm-season grasses including *Wrangler* bermudagrass and *Quick-N-Big* crabgrass. Thus, it is possible that differences in chemical composition, particularly for soluble carbohydrates, would have been more apparent if additional samples had been collected later in the day during grazing events.

### Forage Preference of Grazing Horses

The relative humidity on grazing dates ranged from 64.8% to 73.2% (Table 6). Daily maximum temperatures (28.3–35.0 °C) and average temperatures (21.8–27.7 °C) on grazing event days were above historical averages for the months preceding and following each of the grazing days. The largest difference in daily maximum temperatures between the two days of a single grazing event was 5 °C (30 °C vs. 35 °C) for the third grazing event. The daily maximum temperatures were the same (33.9 °C) between the two days of the first grazing event and were also similar between the two days of the second grazing event (28.3 °C vs. 31.1 °C).

**Table 6.** Weather data from grazing event dates during the 2019 grazing season. Data shown include daily average relative humidity and temperature as well as max temperature on grazing event dates. Monthly historical averages for max and average temperature in July – October are also presented^1^.

Date	Average relative humidity, %	Max temperature, °C		Average temperature, °C	
		2019	Historical average	2019	Historical average
Jul 29, 2019	68.3	33.9	Jul: 29.7 Aug: 28.6	26.9	Jul: 23.8 Aug: 22.8
Jul 30, 2019	69.0	33.9		27.7	
Aug 29, 2019	64.8	28.3	Aug: 28.6 Sep: 25.2	21.8	Aug: 22.8 Sep: 19.2
Aug 30, 2019	69.6	31.1		22.1	
Oct 1, 2019	73.2	30.0	Sep: 25.2 Oct: 19.1	22.5	Sep: 19.2 Oct: 13.0
Oct 2, 2019	69.9	35.0		25.3	

Weather data were obtained for the New Brunswick Station through the Office of the New Jersey State Climatologist website (Rutgers New Jersey Weather Network 2020; https://www.njweather.org/data). As each grazing event was conducted at either the end or beginning of a month, historical monthly averages of the months preceding and following the grazing event date are shown.

**Figure 5.**
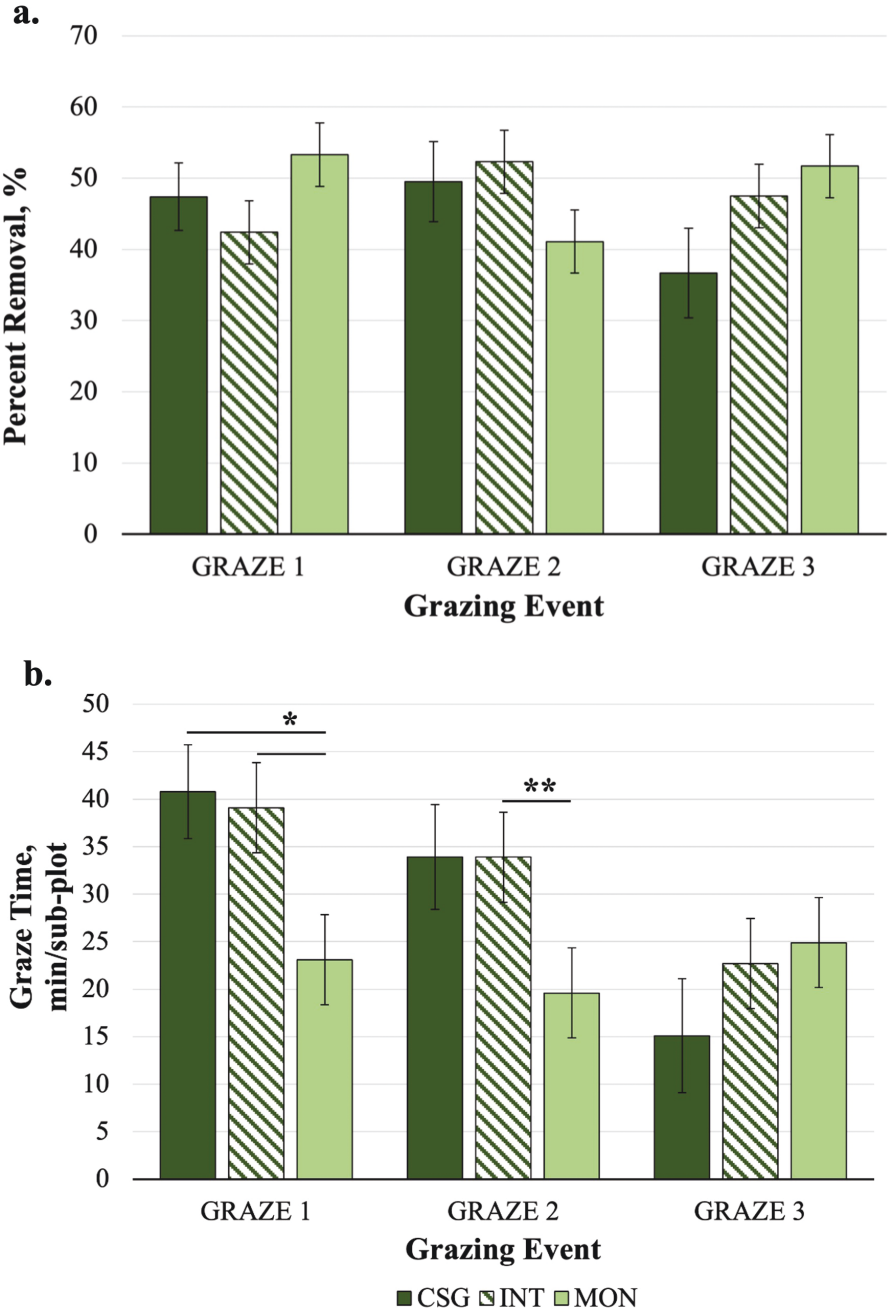
Horse grazing preference variables by treatment [mixed cool-season grass only (CSG), *Quick-N-Big* crabgrass interseeded into existing mixed cool-seson grass (INT), and *Quick-N-Big* crabgrass established as a monoculture (MON)] across three grazing events in 2019: Jul 29/30, 2019 (GRAZE 1), Aug 29/30 (GRAZE 2), Oct 1/2 (GRAZE 3) including (a) percent forage removal (%), and (b) time spent grazing per sub-plot (GT; min/sub-plot). Cool-season grass species in pasture mix included *Argyle* Kentucky bluegrass, *Inavale* orchardgrass, and *Tower* tall fescue. A single asterisk indicates differences between treatments within grazing events at *P ≤* 0.05. Double asterisks indicate a trend for differences *P ≤* 0.10. Data are presented as the means ± SEM.

Percent removal of pasture forage did not differ by either treatment or grazing event, and there was no treatment by grazing event interaction (Fig. 5a). While the SH measurements did not provide conclusive evidence of horse grazing preference, observations of horse grazing activity indicated that horses may prefer cool-season grasses over CRB. The GT varied by treatment and grazing event, and there was a significant treatment by grazing event interaction (*P <* 0.003). Horses spent less time grazing in MON subplots (22.6 ± 3.77 min/subplot) than in INT (31.9 ± 3.79 min/subplot; *P =* 0.003) or CSG (29.9 ± 4.17 min/subplot; *P =* 0.07). Horse GT was greater for GRAZE 1 (34.3 ± 3.82 min/subplot) and GRAZE 2 (29.1 ± 3.91 min/subplot) than GRAZE 3 (20.9 ± 3.99 min/subplot). In pairwise comparisons of the interaction term, GT was lowest for MON in GRAZE 1 vs. INT or CSG (*P ≤* 0.04; Fig. 5b). In GRAZE 2, there was a trend for greater GT in INT compared to MON (*P =* 0.10). Within treatments across grazing events, GT in INT was greater for GRAZE 1 than GRAZE 3 (*P =* 0.03), and GT in CSG was greater in GRAZE 2 vs. GRAZE 3 (*P =* 0.05).

A lesser preference for CRB may actually offer some advantage for management of overweight or obese grazing horses. Ad libitum intake of pasture forage by grazing horses can exceed 3% BW DM per day, and pastured horses may greatly exceed daily caloric requirements leading to weight gain (Smith et al., 2007; Longland et al., 2012). Current feeding recommendations for obese horses include limiting pasture access and dietary NSC intake (Frank et al., 2010). Thus, CRB may thus serve as a potential forage option for these horses. This suggestion agrees with previous findings that horses maintained on CRB from mid-July to mid-September did not gain weight, despite ad libitum pasture access (Weinert-Nelson et al., 2021). While GT has been previously utilized as an indicator of preference (Edouard et al., 2009), it is possible that greater GT could also be attributed to horses spending a greater amount of time selectively grazing (but not necessarily removing a greater amount of forage).

The discrepancy between results for percent removal and grazing time as indicators of preference in this study may be, in part, attributable to methods of measuring forage removal. Prior equine grazing studies have assessed short-term forage removal in restricted grazing areas using pre- and post-grazing measures of herbage mass using either harvested quadrats and/or a falling plate meter (Dowler et al., 2012; Glunk et al., 2013; Jaqueth, 2018). However, this method of assessing pasture forage removal has limitations. Jaqueth (2018) noted that the quadrat method at times resulted in negative values for forage removal. As our lab has also observed negative values for pasture forage removal in previous studies when using herbage mass measured by the quadrat method as the metric, in this study, pre- and post-grazing SH were assessed to determine forage removal. While this approach has the advantage of collecting more observations per subplot (10 data points for SH vs. 3 for HM) without artificially removing/harvesting any of the forage within the subplot, utilizing SH as the metric for assessing removal may also be less than ideal. Pasture SH does not account for forage density (Weinert and Williams 2018). Additionally, in the current study, we observed that CRB was more prone to damage from trampling, and thus the post-grazing SH measurements in these plots likely did not capture the full extent of remaining, un-grazed forage (Ghajar et al., 2021). A number of previous equine grazing studies have used a visual estimation technique to assess percent removal of pasture forage in small plots (Allen et al., 2013; Grev et al., 2017; Catalano et al., 2020). While somewhat subjective, this technique may have better accounted for the impact of forage density and trampling while avoiding the potential for negative removal estimates. A more accurate method for assessing percent removal would have benefitted assessment of horse preference in the current study and would have allowed for a definitive interpretation of GT as a marker of horse forage preference.

Previous studies have found correlations between forage physical and chemical characteristics and forage preference of grazing horses. A negative correlation has been reported between horse preference and pasture forage maturity (McCann and Hoveland, 1991; Catalano et al., 2020) and height (Fleurance et al., 2010; DeBoer et al., 2017; Catalano et al., 2019, 2020). Forage NDF has also been found to be negatively correlated with preference (Allen et al., 2013; DeBoer et al., 2017; Catalano et al., 2020), while CP (Edouard et al., 2010; DeBoer et al., 2017; Catalano et al., 2019, 2020), DE (Catalano 2019, 2020), and NSC (Borgia et al., 2011; Allen et al., 2013; DeBoer et al., 2017) have been positively correlated with horse preference. The current study found no significant correlations between forage nutrients and horse preference as assessed either by forage removal or GT. Percent removal was positively correlated with pre-grazing SH (*r*_s_ = 0.49; *P <* 0.0001). However, a relationship was not found for pre-grazing SH and GT. There was also a weaker positive correlation between percent removal and HM (*r*_s_ = 0.33; *P =* 0.009) and a negative correlation with DM (*r*_s_ = −0.25; *P =* 0.05). Neither of these variables were correlated with GT. In fact, the only relationship between GT and any forage characteristics was a weak negative correlation with maturity (*r*_s_ = −0.22; *P =* 0.003).

It is possible that methods utilized for assessment of forage removal, as discussed above, in addition to pooling of replicate treatment samples for nutrient analysis may have impacted correlation analysis with forage variables. However, prior studies of horse forage preference have emphasized the complex and multifactorial interplay of forage, environmental, and animal characteristics that shape equine feeding behaviors (Marten, 1978; Grev et al., 2017; Catalano et al., 2020). Physical properties of forages such as smell, taste/flavor, touch/coarseness (mouth-feel), and palatability can all affect preference (Marten, 1978; Ellis, 2010; Catalano et al., 2020). Ellis et al. (2010) also highlighted the potential influence of feed fracture properties (and thus chewing efficiency) on feeding behavior and intake. The lack of relationships between forage variables and GT, would suggest that other factors not measured in the current study may have influenced horse preference and grazing behavior. While not measured, it was observed that the stem diameter of CRB tended to be larger than that of cool-season grasses. Additionally, CRB leaves and stems are densely hairy. Such factors could have impacted horse preference as assessed by GT.

## CONCLUSION

Results of this study provide further support for integration of *Quick-N-Big* crabgrass established in monoculture as a strategy to improve cool-season grass pasture production during the “summer slump” period. However, interseeding did not produce similar yields as monoculture establishment and interseeded yield also was not significantly greater than cool-season grasses. Therefore, while interseeded was 32% more than in cool-season pasture grass, it is unlikely that interseeding crabgrass would be effective in increasing summer yields of traditional cool-season equine rotational grazing systems. Both treatments containing CRB provided adequate nutrition to meet requirements of horses at maintenance, but all treatments provided low NSC to grazing horses. Voluntary grazing in all treatments demonstrated that the crabgrass was palatable to grazing horses. However, horses spent more time grazing in treatments containing cool-season grasses, indicating a possible preference for these grasses in comparison to crabgrass. Low preference coupled with low NSC indicate that crabgrass may offer a potential pasture forage for overweight or obese horses.
